# Tea polyphenols induce S phase arrest and apoptosis in gallbladder cancer cells

**DOI:** 10.1590/1414-431X20176891

**Published:** 2018-03-01

**Authors:** Jiaqi Wang, Yixuan Pan, Jiacheng Hu, Qiang Ma, Yi Xu, Yijian Zhang, Fei Zhang, Yingbin Liu

**Affiliations:** 1 High School Affiliated Fudan University, Shanghai, China; 2 Shanghai Research Center of Biliary Tract Disease, Shanghai, China

**Keywords:** Gallbladder cancer, Tea polyphenols, Apoptosis

## Abstract

Gallbladder cancer (GBC) is the most common malignancy in the biliary tract. Without effective treatment, its prognosis is notoriously poor. Tea polyphenols (TPs) have many pharmacological and health benefits, including antioxidant, anti-inflammatory, anti-tumor, anti-thrombotic, antibacterial, and vasodilatory properties. However, the anti-cancer effect of TPs in human gallbladder cancer has not yet been determined. Cell viability and colony formation assay were used to investigate the cell growth. Cell cycle and apoptosis were evaluated by flow cytometry analysis. Western blot assay was used to detect the expression of proteins related to cell cycle and apoptosis. Human tumor xenografts were used to examine the effect of TPs on gallbladder cancer cells *in vivo*. TPs significantly inhibited cell growth of gallbladder cancer cell lines in a dose- and time-dependent manner. Cell cycle progression in GBC cells was blocked at the S phase by TPs. TPs also induced mitochondrial-related apoptosis in GBC cells by upregulating Bax, cleaved caspase-3, and cleaved PARP expressions and downregulating Bcl-2, cyclin A, and Cdk2 expressions. The effects of TPs on GBC were further proven *in vivo* in a mouse xenograft model. Our study is the first to report that TPs inhibit GBC cell growth and these compounds may have potential as novel therapeutic agents for treating gallbladder cancer.

## Introduction

Gallbladder cancer (GBC) is the most common malignancy in the biliary tract and the sixth most common among gastrointestinal cancers (1–3), comprising 80–95% of biliary tract cancers worldwide. It is also the most aggressive biliary tract cancer, characterized by frequent chemo-resistance and recurrences after surgery. Currently, surgery is the most effective method for patients with GBC (4). However, many patients are diagnosed with advanced GBC with cancer cells that have been spread to lymph nodes or distal organs. For advanced stage GBC, surgical intervention is not possible (5), with unfavorable outcomes after chemotherapy or radiotherapy (6). Only 20% of patients respond to 5-fluorouracil, and 36% respond to gemcitabine (7). The prognosis for patients of advanced GBC is poor, with 5-year survival rates from 20 to 40% (8). Therefore, it is important and necessary to find novel and effective therapy for GBC patients.

Tea polyphenols (TPs) are among the main components in green tea, and include epigallocatechin, epigallocatechin-3-gallate, epicatechin, and epicatechin-3-gallate. TPs have many pharmacological and health benefits including antioxidant, anti-inflammatory, anti-tumor, anti-thrombotic, antibacterial, and vasodilatory properties (9). TPs can induce apoptosis in various cancer cells, such as MCF-7 cells (10), bladder cancer SW780 cells (11), hepatoma HepG2 cells (12), and lung cancer H460 cells (13). TPs may mediate tumor cell apoptosis by regulating apoptosis-related proteins and signaling pathways; TPs have been shown to down-regulate NF-κB, caspases, Bcl-2, and stabilize p53 (14). In addition, TPs regulate cancer cell growth, survival, angiogenesis, and metastasis by MAPKs/AP-1 and PI3K/Akt signaling pathway (15,16).

Currently, however, there is a lack of data showing the effects of TPs in human gallbladder cancer. Here, we aimed to evaluate whether TP treatment inhibited the growth of various GBC cell lines.

## Material and Methods

### Drugs and reagents

TPs were obtained from the National Institute for the Control of Pharmaceutical and Biological Products (China). The purity was greater than 95% as determined by high-pressure liquid chromatography. TPs stock solution (40 mM) was prepared by dissolving TPs in dimethyl sulfoxide (DMSO). The stock solution was stored at -20°C. Dilutions of different concentrations were made using fresh complete medium before each treatment. DMSO was used as control. Antibodies against Bax, Bcl-2, Bad, cleaved-PARP, cleaved-caspase 3, and GAPDH were purchased from Cell Signaling Technology (USA). CyclinA and CDK2 antibodies were purchased from Santa Cruz Biotechnology (USA). The antibody against Ki67 was purchased from Abcam (UK).

### Cell culture

The SGC-996, NOZ, OCUG-1, and GBC-SD human gallbladder cancer cell lines were purchased from the Cell Bank of Type Culture Collection of the Chinese Academy of Sciences (China). Normal gallbladder epithelial cells (HGEpCs) were developed from normal gallbladder tissue (17). NOZ cells line was maintained in William's medium (Gibco, USA) supplemented with 10% fetal bovine serum (FBS; Gibco), 100 U/mL penicillin-streptomycin (Hyclone, USA) and glutamine at 4 mM. Dulbecco's Modified Eagle's Medium (DMEM; Gibco) containing 10% FBS was used to maintain SGC-996, GBC-SD, OCUG-1, and HGEpC cell lines. A 37°C humidified incubator with 5% CO_2_ was used to culture all the cell lines_._

### Cell viability assay

Cell viability was evaluated by the tetrazolium dye 3-(4,5-dimethylthiazol-2-yl)-2,5-diphenyltetrazolium bromide (MTT, Sigma-Aldrich, USA). GBC cells were seeded (6×10^3^ cells/well) into 96-well plates and incubated at 37^o^C overnight. Cells were then treated with TPs solutions of different concentrations (0, 70, 140, 210, 280, or 360 μM) for 24, 48, or 72 h. After each treatment time, 10 µL of MTT reagent were added to each well (0.5 mg/mL) followed by 4-h incubation at 37°C. Then the cell culture medium was discarded and supplemented with 200 µL of DMSO. The absorbance at 490 nm was measured immediately using a 96-well plate ELISA reader (Bio-Rad Laboratories, USA).

### Colony formation assay

GBC cells (500/well) were seeded and treated with TPs of different concentrations (0, 70, or 140 μM) for 48 h. After the treatment, the cells were maintained in culture medium for 10 days, allowing colony formation. The colonies were then fixed with 4% paraformaldehyde, followed by 0.1% crystal violet (Sigma-Aldrich) staining. The colonies were photographed using a microscope (Leica, Germany) and random fields were chosen for colony counting.

### Cell cycle analysis

After being treated with TPs of different concentrations (0, 70, or 140 μM) for 48 h, GBC cells were trypsinized and fixed in 70% ethanol at 4°C overnight. PI staining followed by flow cytometry analysis on a FACS Calibur system (Becton Dickinson, USA) was used to examine cell cycle. At least 50,000 stained cells for each sample were subjected to the analysis each time.

### Hoechst 33342 staining

After being treated with TPs of different concentrations for 48 h, the cells were washed in PBS and stained with 5 μg/mL Hoechst 33342 (Sigma-Aldrich) at 37°C in the dark for 10 min. Nuclear DNA staining was observed using a fluorescence microscope. In each group, five microscopic fields were randomly selected and nuclear staining was counted.

### Annexin V/PI analysis

GBC cells were treated with TPs of different concentrations (0, 70, or 140 μM) for 48 h. Apoptotic rate of cells was analyzed using an Annexin V/PI apoptosis kit (Invitrogen, USA). Staining and measuring procedures were performed according to the manufacturer's protocol.

### Mitochondrial membrane potential (ΔΨm) assay

Fluorescent dye rhodamine 123 probe (Sigma-Aldrich) was used to analyze the mitochondrial membrane potential. After being treated with TPs of different concentrations for 48 h and washed twice with PBS, the cells were incubated with Rhodamine 123 (5 μg/mL) for 30 min at 37°C. The final analysis was done by flow cytometry (Becton Dickinson).

### Western blot analysis

After the treatment with TPs of different concentrations (0, 70, or 140 μM) for 48 h, GBC cells were harvested and total cellular proteins were extracted for western blot analysis. Protein concentrations were measured by BCA kit (Beyotime, China). Equal amounts of protein (50 ng) were loaded into 12% SDS-polyacrylamide gel for electrophoresis (SDS-PAGE). Nitrocellulose membranes were used for electro-transfer, which was performed at 300 mA for 2 h. Five percent skim milk in Tris-buffered solution containing 1% Tween-20 (TBST) was used to block the membrane. Membranes were then incubated with corresponding primary antibodies according to the protocols, followed by incubation with secondary antibodies at room temperature for 1 h. A chemiluminescence (ECL) detection system was used for exposure.

### *In vivo* efficacy of TPs

Six-week old BALB/c homozygous (nu/nu) nude mice (body weight of about 18 g) were purchased from Shanghai SLAC Laboratory Animal Co., Ltd. (China). Tumor inoculation was performed in mice of 7 weeks of age. NOZ cells (2×10^6^) suspended in 100 µL PBS were injected into the right flank subcutis of the nude mice. The mice were randomly divided into 3 groups (4 mice/group): PBS (control), 20, or 40 mg/kg TPs were given to each group, respectively, via intraperitoneal (*ip*) injection every day for 5 weeks. After the 5-week treatment, the mice were sacrificed and tumors were removed to determine the tumor sizes and weights. All animal procedures were conducted under guidance of the Care and Use of Laboratory Animals from National Institutes of Health. The animal protocol was approved by the Institutional Animal Care and Use Committee of Shanghai, Jiaotong University.

### Statistical analysis

All results are reported as means±SD, calculated from at least three independent experiments. Differences between groups were analyzed using one- or two-way ANOVA with Dunnett's *post hoc* test. A P value of less than 0.05 was considered to be significant. SPSS software, v19.0 (SPSS Inc., USA) was used for all statistical analyses.

## Results

### TPs inhibited GBC cell growth in a dose- and time-dependent manner

**Figure 1.**
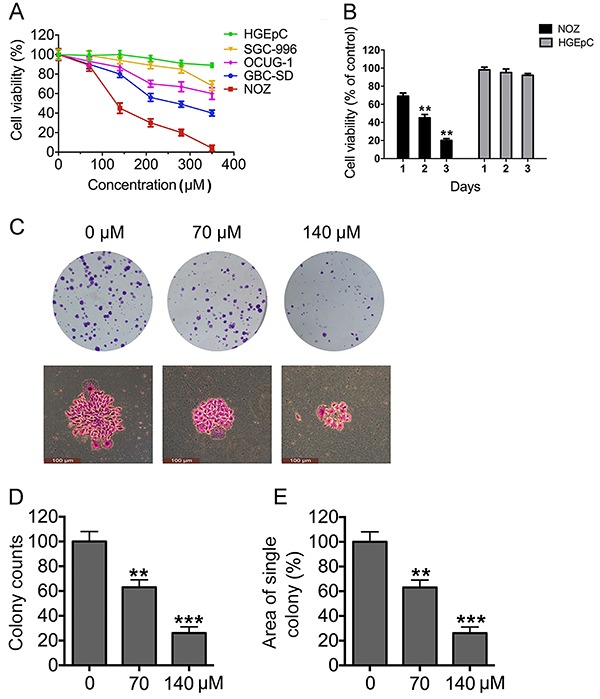
Tea polyphenols (TPs) inhibit gallbladder cancer (GBC) cell growth in a dose- and time-dependent manner. *A*, Four GBC cells and one normal gallbladder epithelial cell were assayed for cell viability after exposure to TP concentrations that ranged from 0 to 350 μM; untreated cells were used as controls. All the cells were cultured for 48 h. *B*, At a fixed dose (140 μM), TPs inhibited the viability of four GBC cells and one normal gallbladder epithelial cell in a time-dependent manner (24, 48, or 72 h). *C*, Colony formation assays were performed using NOZ cells treated with indicated doses of TPs. The colonies were stained with crystal violet. *D*, Colony formation was counted manually for each group of cells. *E*, The size of each colony was also calculated and averaged. **P<0.01; ***P<0.001 compared to 0 (ANOVA).

The result showed that TPs had a dose- and time-dependent killing effect on multiple GBC cell lines, but not on HGEpCs (Figure 1A and B), indicating that TPs selectively kill GBC cells but not normal cells. The NOZ cell line was the most sensitive to TPs among four cell lines; TPs showed a significantly higher toxic effect on this cell line, with an IC_50_ value around 100 μM. Therefore, NOZ cell line was chosen for further functional analyses. NOZ cells were treated with TPs at concentrations from 0 to 140 μM *in vitro*. Furthermore, we adopted the doses 0, 70, and 140 μM of TPs due to the initial cytotoxicity results and subsequently used these concentrations to perform colony formation assays. It was visually observed that smaller colonies were formed as the TP dosage increased (Figure 1C). Quantitative analysis indicated that colony numbers decreased with increased TP dosage (Figure 1D). A smaller average growth area of a single colony was also observed with an increasing TP dosage (Figure 1E). These results suggest that TPs inhibit NOZ cell growth in a dose- and time-dependent manner.

### TPs induced S phase arrest in GBC cells

**Figure 2.**
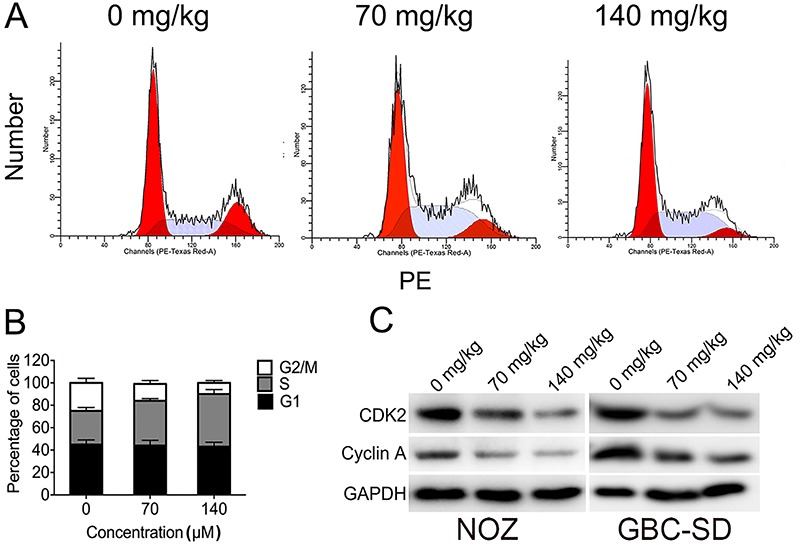
Tea polyphenols (TPs) induce S phase arrest in gall bladder (GBC) cells. *A*, *B*, NOZ cells without or with different TP treatment were subjected to cell cycle analysis by flow cytometry. *C*, Western blot analysis of cell cycle-related proteins in both cell lines. GAPDH was used as a loading control. Data are reported as means±SD of three independent experiments.

TP treatment arrested the cell cycle in NOZ cells. More importantly, the percentage of cells in the G2/M phase decreased as the TP dosage increased. In contrast, there was an increased percentage of cells in the S phase in TP-treated NOZ cells, compared to controls (Figure 2A). The results suggested that TPs can arrest the cell cycle at the S phase in a dose-dependent manner. The result was further confirmed by western blotting, showing that S-G2/M related proteins cyclin A and CDK2 were down regulated in NOZ and GBC-SD cells after TP treatment (Figure 2B), consistent with the TP-induced S phase arrest. These results suggest that TPs induced S phase arrest by regulating S phase-related proteins cyclin A and CDK2 in GBC cells.

### TPs triggered mitochondrial-related apoptosis of GBC cells

**Figure 3.**
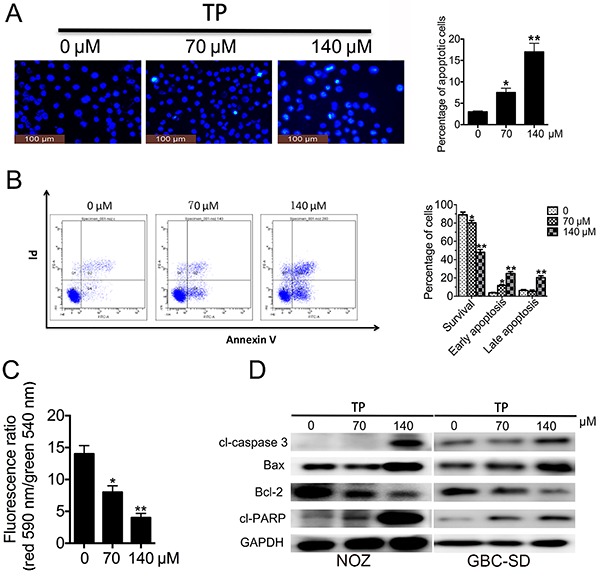
Tea polyphenols (TPs) trigger mitochondrial-related apoptosis of gallbladder cancer (GBC) cells. *A*, Changes in apoptotic nuclear morphology were observed by Hoechst 33342 staining and visualized by fluorescent microscopy. *B*, Cells were analyzed by flow cytometry with Annexin V-FITC/PI staining after TP treatment. Annexin V *vs* PI plots from the gated cells show the populations corresponding to viable (Annexin V-/PI-), necrotic (Annexin V-/PI+), early (Annexin V+/PI-), and late (Annexin V+/PI+) apoptotic cells. *C*, Flow cytometric analysis of the mitochondrial membrane potential (DΨm). The percentages of cells with low DΨm are shown. *D*, Western blot analysis of apoptosis-related proteins in both cell lines; GAPDH was used as a loading control. Data are reported as means±SD of three independent experiments. *P<0.05, **P<0.01 *vs* control (ANOVA).

Chromatin condensation and fragmentation are two major indicators of cell apoptosis. In order to study the role of apoptosis in TPs mediated anti-cancer activities, we studied the nuclear morphology of TP-treated cells using Hoechst 33342 staining. The nuclei were stained homogeneously and weakly in blue in control cells, whereas some nuclear fragmentation and bright chromatin condensation were observed in TP-treated cells (Figure 3A). The amount of apoptotic nuclei indicated by bright chromatin condensation increased significantly as the TP concentration increased (Figure 3B). Apoptosis was confirmed by flow cytometry analysis of Annexin V-FITC/PI staining. As shown in Figure 3B, apoptosis in NOZ cells was increased at both early and late stages in a dose-dependent manner after TP treatment. These results strongly suggest that TP-induced inhibition of cell growth coincides with increased apoptosis in GBC cells. To further determine whether the apoptosis was mitochondria-dependent, we examined the mitochondrial membrane potential in TPs treated cells. Compared to controls, TP treatment caused an obvious, dose-dependent decrease in the mitochondrial membrane potential in NOZ cells (Figure 3C). Apoptosis is a caspase-dependent programmed cell death; a significant proteolytic cleavage of caspase-3 and PARP were observed in TPs treated NOZ cells. Furthermore, the level of pro-apoptotic mitochondrial protein Bax was increased, whereas the level of anti-apoptotic protein Bcl-2 was decreased in TPs treated NOZ and GBC-SD cells (Figure 3D). These results indicate that TPs can induce apoptosis through mitochondrial signaling pathways in GBC cells.

### TPs inhibited tumor growth in a xenograft model of GBC

**Figure 4.**
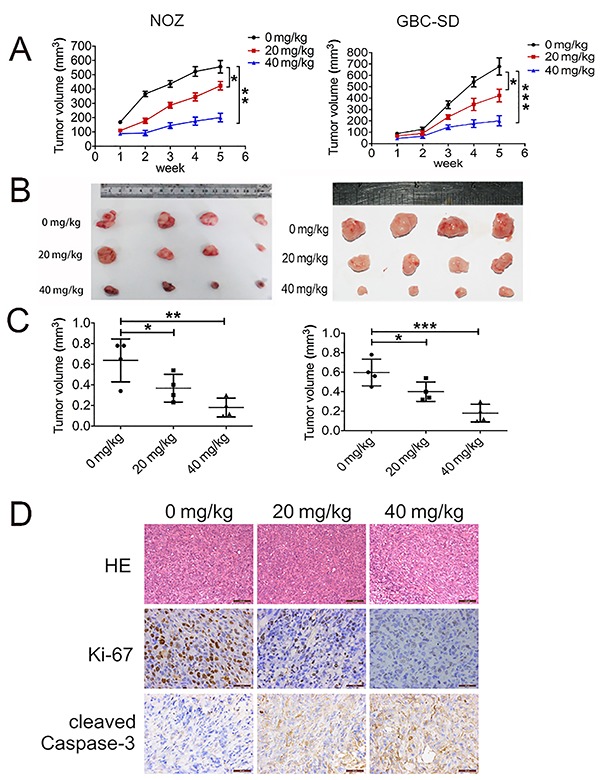
Tea polyphenols (TPs) effect on tumor growth in a xenograft model of gallbladder cancer (GBC). *A*, Volumes of dissected tumors during 6 weeks. *B*, Photographs of dissected tumors from each group after 7 weeks. *C*, Comparison of tumor volumes between groups. *D*, Expression of Ki-67 and cleaved-Caspase-3 was detected in xenografts by IHC. Data are reported as means±SD. *P<0.05; **P<0.01 compared to control (ANOVA).

A xenograft model of GBC was established using NOZ and GBC-SD cells. Mice were injected with TPs (20 or 40 mg/kg) daily for 5 weeks; mice that were injected with PBS were used as controls. The tumor volumes were different three weeks after inoculation. After five weeks, the mice in the control group exhibited the largest tumor volumes, whereas mice treated with TPs exhibited significantly smaller tumor sizes than the control group (Figure 4A). After dissection, the tumor sizes were significantly larger in control mice than TP-treated mice (Figure 4B). Tumor weights were significantly different among the three groups as well. The average weight of tumor in mice injected with 20 and 40 mg/kg of TPs was only 65.4 and 56.7% of that in control mice, respectively. Treatment with 40 mg/kg of TPs remarkably shrunk the tumors compared to those in control group (Figure 4C). Furthermore, IHC staining showed decreased levels of proliferating cell nuclear antigen (Ki67), indicating significantly fewer proliferative cells, and increased levels of cleaved caspase-3, indicating significantly more apoptotic cells in TP-treated xenograft tumors than in control tumors (Figure 4D). These results suggest that TPs can inhibit GBC growth *in vivo*.

## Discussion

GBC is the 6th-most common digestive tract malignancy worldwide (18). There is lack of effective therapy available for advanced GBC at present, and it is important to discover new therapies. Chinese herbal medicines have been shown to have effective antitumor activities with minimal toxicities. Therefore, they could be valuable candidates for developing novel chemotherapeutic agents.

TPs suppress tumors by inhibiting cell proliferation (19) and promoting tumor cell apoptosis (20). Targeting the molecules involved in apoptosis may provide new ideas for the search and development of anti-tumor drugs. TPs were involved in regulating a variety of signaling pathways in apoptosis in tumor cells. TPs inhibit the activity of protein kinase C and CDKs, upregulating the expression of IkBa, and initiating caspase cascades in mitochondria (21). In addition, TPs exert their chemoprevention effects by blocking proliferation and differentiation through modulating EGFR-MAPK signaling pathway and regulating c-jun, c-fos, and c-myc expression (22).

In this study, GBC cell growth was significantly suppressed by TPs both *in vivo* and *in vitro*. We further examined the effects of TPs on apoptosis and cell cycle arrest in GBC cells, since they are two major indicators of anticancer activities in cancer cells. The results suggested that TPs could arrest GBC cell cycle at S phase and induce its mitochondria-related apoptosis. Moreover, the results showed that TPs had no toxic effects on normal gallbladder cells. Therefore, our results indicated that TPs is a safe molecule with a significant potential for GBC treatment clinically.

Apoptosis is a programmed cell death, which plays a critical role in development and health maintenance (9). Mitochondria-related apoptosis is the major apoptotic pathways in cancer cells (23). A study showed that TPs mediated tumor cell apoptosis by regulating apoptosis-related proteins and signaling pathways. TPs have been shown to down-regulate NF-κB, caspases, and Bcl-2, and stabilize p53 (14). In addition, TPs regulate cancer cell growth, survival, angiogenesis and metastasis by MAPKs/AP-1 and PI3K/Akt signaling pathway (15,16). TP also promoted apoptosis in cancer cells in both the presence and absence of p53 function, through the survival signaling pathways that converge in the execution of apoptosis through involvement of the mitochondrial death cascade (19).

In our study, we found that treating NOZ cells with TPs for 48 h decreased the mitochondrial membrane potential, suggesting that the mitochondrial-related apoptosis played an important role in TPs induced apoptosis of gallbladder cancer cells. To further explore the mechanism of the TP-induced mitochondrial-related apoptosis, we detected the cell expression levels of Bcl2, Bax, cleaved caspase-3, and cleaved PARP. Our data showed that Bax expression was increased and Bcl2 expression was decreased after TP treatment. The change of the protein expression profile suggested a reduction in mitochondrial membrane potential and an increase in cytochrome c releasing into the cytosol as a result. Our results also showed that TPs treatment can increase the expression of cleaved caspase-3 and cleaved PARP. These proteins belong to the Caspases family, which is involved in the downstream events of mitochondrial-mediated apoptosis (24,25). Our results suggested that TPs treatment induces mitochondrial-mediated apoptosis by activating the caspase-3 and PARP apoptotic cascade. TPs-induced apoptosis in GBC cells occurred through the mitochondrial pathway.

Taken together, the current study provides for the first time evidence that TPs can induce cell cycle arrest and promote apoptosis in GBC cells. The anticancer activities of TPs suggest that they are a promising candidate for the development of novel therapeutic reagents against GBC.
